# Cannabinerol and NSC-34 Transcriptomic Analysis: Is the Dose Who Makes Neuronal Differentiation?

**DOI:** 10.3390/ijms23147541

**Published:** 2022-07-07

**Authors:** Andrea Valeri, Luigi Chiricosta, Agnese Gugliandolo, Federica Pollastro, Stefano Salamone, Valeria Domenica Zingale, Serena Silvestro, Emanuela Mazzon

**Affiliations:** 1 IRCCS Centro Neurolesi “Bonino-Pulejo”, Via Provinciale Palermo, Contrada Casazza, 98124 Messina, Italy; andrea.valeri@irccsme.it (A.V.); luigi.chiricosta@irccsme.it (L.C.); agnese.gugliandolo@irccsme.it (A.G.); valeria.zingale@irccsme.it (V.D.Z.); serena.silvestro@irccsme.it (S.S.); 2 Department of Pharmaceutical Sciences, University of Eastern Piedmont, Largo Donegani 2, 28100 Novara, Italy; federica.pollastro@uniupo.it (F.P.); stefano.salamone@uniupo.it (S.S.); 3 PlantaChem Srls, Via Amico Canobio 4/6, 28100 Novara, Italy

**Keywords:** cannabinoids, phytocannabinoids, cannabinerol, NSC-34, neurogenesis, neuronal differentiation

## Abstract

*Cannabis sativa* L. proved to be a source of several phytocompounds able to help patients facing different diseases. Moreover, these phytocompounds can help ameliorate general conditions and control certain unpleasant effects of diseases. Some cannabinoids, however, provided more benefits applicable to settings other than palliative care. Using the NSC-34 cell line, we evaluated the barely known phytocompound named cannabinerol (CBNR) at different doses, in order to understand its unique characteristics and the ones shared with other cannabinoids. The transcriptomic analysis suggests a possible ongoing neuronal differentiation, principally due to the activation of cannabinoid receptor 1 (CB1), to which the phosphorylation of serine–threonine protein kinase (Akt) followed, especially between 20 and 7.5 µM. The increase of *Neurod1* and *Map2* genes at 7.5 µM, accompanied by a decrease of *Vim*, as well as the increase of *Syp* at all the other doses, point toward the initiation of differentiation signals. Our preliminary results indicate CBNR as a promising candidate to be added to the list of cannabinoids with neuronal differentiation-enhancer properties. However, further studies are needed to confirm this initial insight.

## 1. Introduction

In 1988, a spark of knowledge enlightened the field of *Cannabis sativa* L. research: the first study investigating the endocannabinoid system was performed which subsequently led to the growth of an entire field of research [1]. Currently, the word “cannabinoids” recalls more than 20,000 articles on Pubmed since the 1988 discovery. *C. sativa* is a plant well known to humankind, and indeed its use can be dated back to Ancient Egypt, and the Fifth Dynasty, around 2300 years before Christ [2]. The first article on PubMed regarding the use of cannabis for medicinal purposes dates back to 1840 [3].

**Figure 1:**
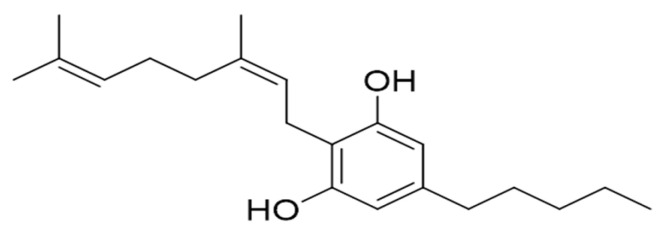
Chemical structure of cannabinerol (CBNR).

Over 500 compounds have been identified from *C. sativa* and over 100 are cannabinoids [4], where Δ^9^-tetrahydrocannabinol (Δ^9^-THC) and cannabidiol (CBD) are the most abundant and famous. Both of them possess the capacity to relieve pain and anxiety, with the important difference that THC also bears psychoactive effects [5]. Of course, there are many other cannabinoids and one of the least known is cannabinerol (CBNR), whose chemical structure is shown in Figure 1.

The first hint of its existence came from the isolation of its precursor, cannabinerolic acid, which was thought to be part of the biosynthesis cascade of Δ^1^-tetrahydrocannabinolic acid, using chromatography of air-dried cannabis leaves [6]. Due to its chemical structure, it is categorized into the subclasses of cannabigerol (CBG)-like cannabinoids [7], since cannabinerolic acid is the *trans*-isomer of the cannabigerolic acid (CBGA) [8]. A more precise definition of CBNR came in 2016, when chemical analysis of an Indian hashish sample was performed, which allowed the scientific community to obtain the mass spectrum of this unknown compound [9].

The available information on CBNR ends here. Because of the current lack of information regarding this compound and thanks to its similarity to CBG, a cannabinoid already known for its neuroprotective and neuroregenerative properties, we decided to perform an in vitro test to evaluate its effects on neurons.

Despite the fact that *C. sativa*’s properties have been a matter of study for a long time, the scientific community started to consider the idea of neurogenesis induced by phytocannabinoids only recently.

The concept of neuronal irreplaceability was placed in doubt long ago, from the first evidence of adult neurogenesis in rats in 1965 [10], and subsequent different studies aimed at understanding the location and mechanisms of neurogenesis in the adult mammal brain. It is now known that neurogenesis in adults indeed occurs owing to cells named Neural Progenitor/Stem Cells (NSCs), located in the subventricular zone (SVZ) and the subgranular zone (SGZ) of the hippocampus. They seem to have the capacity to differentiate either in neurons or in astrocytes, while the generation of oligodendrocytes was evaluated in vitro [11]. Since the endocannabinoid system, the complex mechanism of receptor signaling and signal cascade primarily targeted by cannabinoids, also influences the mitogen-activated protein kinases (MAPKs) and mammalian target of rapamycin (mTOR), involved in the stem cell fate signal [12], the next question was whether cannabinoids can also have a role in directing NSCs toward their final differentiating state.

Of course the answer was positive and a large number of experiments were performed, indicating that both cannabinoid receptor 1 (CB1) and cannabinoid receptor 2 (CB2) play an important role in enhancing neurogenesis and triggering neuronal differentiation in cells and animal models [13]. Moreover, our research group previously indicated that CBG could upregulate genes involved in the neuronal regeneration in an in vitro model of spinal cord injury (SCI) when the compound was administered post-injury [14]. CB1 and CB2, however, are not the only receptors involved in endocannabinoid system: the peroxisome proliferator-activated receptor-γ (PPARγ) was also reported to be a target of cannabinoids, such as CBD and CBG [15], and indeed it plays a role both in neurogenesis and differentiation. It has been shown that selective activation of PPARγ after CBD administration significantly increases neurogenesis in the hippocampus of a rat model of Alzheimer’s Disease, along with exerting anti-inflammatory and neuroprotective effects [16]. Regarding differentiation, the endocannabinoid anandamide (AEA) proved to be efficient in the differentiation of NSCs [17] while VCE-003.2, the synthetic cannabigerol derivative proved to be able to induce a neuronal-like differentiation in P19 cells [18]. Another cannabinoid, cannabichromene (CBC), seems to have an effect on NSCs, in particular to inhibit their differentiation toward astroglia, indicating that CBC might assist neuronal differentiation [19].

Using an NSC-34 cell line in an undifferentiated state, we administered CBNR at different concentrations and, after evaluating its possible toxicity, we performed a transcriptomic analysis in order to understand whether, as with CBG, CBNR can also be fitted in the group of the neuronal differentiation-enhancer cannabinoids.

## 2. Results

### 2.1. MTT Test

**Figure 2:**
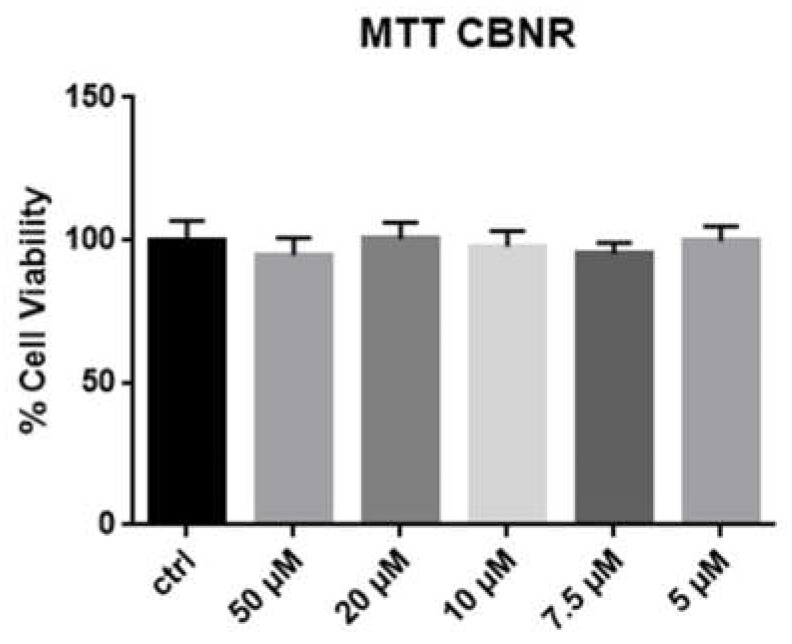
Results of cell viability after incubating NSC-34 cells with different concentrations of CBNR. MTT test indicates that CBNR is not toxic to cells after 24 h of incubation.

In order to investigate the potential toxicity of CBNR to the cells, we incubated NSC-34 for 24 h with a medium containing CBNR at different concentrations: 50, 20, 10, 7.5 and 5 µM. As can be seen from Figure 2, there is no significant difference in cell viability after MTT test. The incubation with the same concentration of dimethyilsulfoxide (DMSO) proved that no cytotoxicity due to this substance was present.

### 2.2. Transcriptomic Inspection

The transcriptomic analysis was performed for each dose of CBNR against the CTRL. In detail, we compared the CTRL against CBNR5, CBNR7.5, CBNR10, CBRN20, CBNR50. The comparison against CBNR50 showed 3788 Differentially Expressed Genes (DEGs), 1844 upregulated and 1944 downregulated. CTRL against CBNR20 revealed 2805 DEGs where 1378 are upregulated and 1427 are downregulated. The CBNR10 has 2168 upregulated, 2069 downregulated and in total 4237 DEGs. On the other hand, the comparison with CBNR7.5 showed 6211 DEGs, 3093 upregulated and 3118 downregulated. The analysis against CBNR5 revealed 4142 DEGs among which 2064 are upregulated and 2078 are downregulated. Since we are interested in the neurogenesis properties of CBNR, genes involved in neurogenesis and neuronal differentiation pathways were inspected, as shown in Table 1 and Table 2.

### 2.3. Western Blot

**Figure 3:**
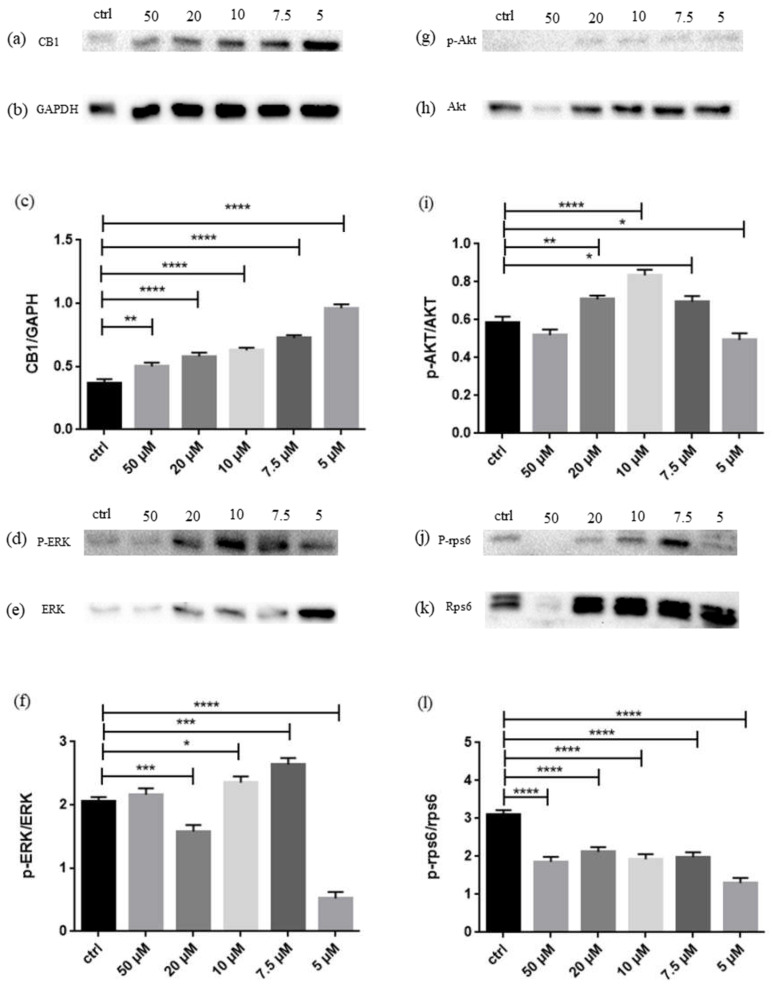
(**a**) Evidence of significant increase of CB1 in NSC-34 after treatment with CBNR; (**b**) GAPDH used for normalization; (**c**) densitometric analysis of CB1, ** *p* < 0.005. **** *p* < 0.0001; (**d**) Evidence of different phosphorylation degree of ERK at different doses; (**e**) not-phosphorylated ERK used for normalization; (**f**) densitometric analysis of p-ERK, * *p* < 0.05. *** *p* = 0.0001. **** *p* < 0.0001; (**g**) Evidence of different phosphorylation degree of Akt at different doses; (**h**) non-phosphorylated Akt used for normalization; (**i**) densitometric analysis of p-Akt, * *p* < 0.05. ** *p* < 0.005. **** *p* < 0.0001; (**j**) Evidence of different phosphorylation degree of rps6 at different doses; (**k**) non-phosphorylated rps6 used for normalization; (**l**) densitometric analysis of p-rps6, **** *p* < 0.0001.

In order to confirm the activation of CB1 and to investigate the activity of Akt, ERK and Rps60, Western Blot analyses were performed. As shown in Figure 3, CB1 level increases at every dose, while phosphorylation of Akt and ERK peaks at 10 µM and 7.5 µM, respectively. The phosphorylation of Rps6 decreases at every dose.

## 3. Discussion

NSC-34 cells are a cell line obtained by the hybridization of embryonic spinal cord cells and neuroblastoma cells from mice. They are enriched with motor neurons, so this cell line could be used as a model for a developing motor neuron system [20]. We used it in an undifferentiated state in order to understand whether our substance of analysis, CBNR, can influence the neurogenesis process. To our knowledge, this is the first time that CBNR has been administrated in an in vitro model: since NSC-34 cells are a model of developing neurons and cannabinoids proved to be useful in neurogenesis, we tried to understand whether CBNR can play a role in the generation of new cells or in differentiation and which receptor could be involved in such process.

After exposing the cells to different concentrations of CBNR, we assessed their viability and concluded that the substance is not toxic, at least until reaching the concentration of 50 µM for 24 h, as Figure 2 shows. After assuring the safety of CBNR, a transcriptomic analysis followed.

Cannabinoids proved to be able to induce neurogenesis and neuronal differentiation, so the classical markers of these processes were inspected. We clearly did not expect to see a full shift of our cells to a mature-like stage, since normally it takes days of exposure to retinoic acid to induce a complete motor neuron-like phenotype [21]. However, initial signals and activation of genes involved in neuronal differentiation might be already present. Nestin, encoded by *Nes*, as well as Vimentin, encoded by *Vim* gene, are markers of neuronal progenitor cells [22] and, for this reason, the decrease of *Vim* at 50, 20 and 7.5 µM pointed toward a differentiating state of the cells. A 7.5 µM concentration of CBNR is the only dose that activates two important markers of neuronal differentiation, Neurod1 [23] and Map2. It is important to mention that MAP2 is one of the markers used to identify differentiated NSC-34 [21]. On the other hand, the other dose seemed to increase the expression of Synaptophysin, encoded by the gene Syp, which is a marker widely used to identify synapses [24] and mature neurons, that decreases only at 7.5 µM. This evidence suggests that neuronal differentiation signals are active, so the possible pathways that CBNR can activate to induce this change were investigated.

CBNR is similar to CBG, thus it is reasonable to think that it may act on the same receptors. As can be seen from Table 2, every dose of CBNR upregulates *Cnr1* gene, encoding for CB1 [25]. Western Blot analysis shown in Figure 3a provided us another indication about CB1: it seems that increasing the dose decreases the amount of receptor, even though it always remains above the level of the control. This is likely due to the saturation and subsequent desensitization of the receptor, which might be internalized and degraded when the dose of the substance—and consequently the stimulation of the receptor—increases. NSC-34 cell line does not express *Cnr2*, so no conclusion could be given about a possible affinity and interaction between CBNR and the Cannabinoid Receptor 2 (CB2), encoded by the last-mentioned gene [26].

CB1 stimulation can lead to neuronal differentiation using Akt pathway [17]. Since the activity of Akt is measured in its phosphorylation, Western Blot was performed and the peak of activity, as demonstrated in Figure 3, seems to be at 10 µM. According to previous evidence [17], the phosphorylation of ERK should decrease during differentiation, thus this part of Akt pathway was also inspected and, as shown in Figure 3, phosphorylation of ERK seems to decrease at 20 and 5 µM, while it is surprisingly high at 7.5 µM. The almost constant downregulation of the substrate of mTOR, the ribosomal protein S6, identified by the gene Rps6, indicates that the mTOR pathway also seems not to be followed. This is in accordance to previous observation regarding neuronal differentiation mediated by CB1, since its phosphorylation seems to also be constantly under the level of the control, as seen in the Western Blot reported in Figure 3 [17]. It is possible that different doses trigger neuronal differentiation with a strength proportional to the concentration. A dose of 5 µM seems too low to initiate complete neuronal differentiation signals in only 24 h and the high level of CB1 may indicate that the receptor is stimulated by the presence of CBNR, but the activity of Akt is just below the level of control. However, CB1 already bears an effect on p-ERK, since it is strongly inhibited and this might represent a very early stage of differentiation. Other evidence suggests that the cells exposed to 5 µM are at early stage of differentiation: it emerges that *Cdc42*, encoding for Cell Division Cycle 42, plays an important role during development, during a knockdown experiment *Cdc42* demonstrated to be important during the early development of dendritic spines [27]. The fact that 5 µM of CBNR upregulates *Cdc42* suggests that at this dose the cells are at an early stage of development toward the mature neuron stage. *Rbfox2*, essential for the proper development of motor function [28], is upregulated at 5, 7.5 and 10 µM, so it will reinforce the evidence of development ongoing. There is another signal that plays an important role in the early stage of neuronal specification: this is calcineurin (CaN), encoded from the genes *Ppp3ca, Pppcb* and *Ppp3cc*. CaN is essential for neuronal induction and thus is required during the early specification of neuroectoderm as a result of antagonizing BMPs signaling [29]. As can be seen from Table 1, *Pppcb* is overexpressed at 5, 7.5 and 10 µM and decreases at the higher dose, 50 µM, as though its activity is no longer necessary. *Cdk5r2* is a gene that encodes p39, a protein that influences the activity of CDK5, involved in neurodevelopment along with p35, encoded by the gene *Cdk5r1* [30]. However, it is important to mention that p35 can be cleaved in p25 by Calpain, encoded from the gene *Capn1*, that in our experimental sets is upregulated at all doses except 10 µM. p25 is more stable and induces overactivation of CDK5, which is detrimental to its deactivation. In particular, it seems able to lead to neurodegeneration [31]. What is important to notice is that upregulation of p39 was found in cortical differentiating neurons, being more resistant to calpain cleavage, while p35 expression remained steady. The role of p39 in development lies in the capacity to induce axonal extension [32] and in our analyses there was no upregulation of *Cdk5r2* at 5 µM, while it started to be upregulated from 7.5 µM until 50 µM. A pathway whose blocking is correlated with neuronal differentiation is Notch1/Rho-associated protein kinase (ROCK) pathway: inhibition of Notch1 through knock-out resulted in neuronal differentiation in NSC-34 and blocking of its downstream effector ROCK1 promoted, in addition to differentiation of the progenitors [33]. In our experiment, *Rock1* is downregulated at 10, 20 and 50 µM, as if the increasing dose could in some way trigger its downregulation. Again, it can be seen that treating the cells with 5 µM of CBNR does not downregulate *Rock1*. The second isoform of ROCK is ROCK2, encoded from the gene *Rock2*, which also showed a kind of dose-dependent trend to downregulation, starting from upregulation at 5 µM and decrease to downregulation at 50 µM. It is involved in different processes, such as apoptosis since it can influence the activity of caspase-3, but our interest is focused on its downregulation that correlates with positive axon outgrowth. This capacity seems mediated by *Limk1*, encoding for LIM Domain Kinase 1, which is downregulated at 5, 7.5 and 10 µM, not deregulated at 20 µM and upregulated at 50 µM [34]. *Rock2* and *Limk1* seem to follow a kind of dose-dependent trend, where increasing the dose seems to increase the expression of *Limk1*, allowing it to interact with *Rock2.* Another piece of evidence regarding the capacity of CBNR to stimulate differentiation lies in the downregulation of *Camk2b* at all doses. The downregulation of this gene, encoding for Calcium/Calmodulin dependent protein kinase II beta (Camk2β), was correlated with an increase in length of dendrites and an increased arborization of neurites, indicating the role of Camk2β in pruning of neurites [35]. It is important to mention that pruning is equally as important as growth. However, at such an early stage there is no need to selectively retract or remodel neuronal branches. 

CB1, using Akt pathway, could also activate Cyclic AMP-Responsive Element-Binding Protein 1 (CREB) in order to promote the synthesis of brain neurotrophic factor (BDNF) [36], known to promote neuron survival [37]. *Bdnf* is upregulated at 50 and 20 µM. BDNF is also known to promote cannabinoid-mediated differentiation in neurons [38].

All these results suggest that CBNR has the capacity to induce neuronal differentiation of NSC-34 cells using CB1 receptor and Akt pathway. This effect appears to be dose-dependent, since the signals seem to follow a trend where higher doses can trigger more signals than lower doses. However, it has to be pointed out that, once 20 µM of CBNR is reached, we do not observe a faster or more efficient differentiation: at 50 µM there is no expression of more mature neuron markers than at 20 µM. The dose of 20 µM is where the simultaneous increase of Akt phosphorylation and decrease of ERK phosphorylation can be observed, according to the CB1-mediated differentiation [17]. *Ppp3cb, Cdc42* and *Rbfox2*, signals that are usually expressed at the early stages of development, are present below 20 µM, as though this dose might have already pushed the cells to a more mature stage than the lower doses, as suggested by the highest expression of *Syp*.

It can be concluded that CBNR can induce neuronal differentiation in NSC-34 cells after only 24 h and the optimal dose appears to be 20 µM. Lower doses can still trigger neuronal differentiation, but 20 µm seems to demonstrate the most efficient balance between the different signals.

## 4. Materials and Methods

### 4.1. Obtaining CBNR from C. sativa

The cannabinoid cannabinerol was provided by PlantaChem Srls (Novara, IT) with a purity of 98%.

### 4.2. NSC-34 Colture and Treatment

Cedarlane Corporation (Burlington, ON, Canada) was the provider of NSC-34 cell line, providing also the datasheet of the cell line. The maintenance medium was reported to be composed as follows: DMEM High Glucose, 10% Fetal Bovine Serum, 1% penicillin/streptomycin, and 1% L-Glutamine. All reagents were bought from Sigma-Aldrich, Merck KGaA (Darmstadt, Germany).

For MTT test, the cells were seeded in 96-well plates. Next, cells were seeded in 6-well plates, in order to obtain enough cells for transcriptomic analyses and Western Blot analyses. After 24 h of seeding, the maintenance medium was replaced with a same composition medium but with CBNR in different concentrations. The concentrations used were 50, 20, 10, 7.5 and 5 µM in DMSO. One well per plate was used as a control so the medium was simply replaced with a fresh one without CBNR. After 24 h exposure to CBNR, the 96-well plates underwent the MTT test, while the cells in 6-well plates were harvested and centrifuged to obtain the pellet used for the next analyses.

### 4.3. MTT Test

After 24 h treatment with different doses of CBNR, the medium was replaced with a fresh medium with MTT at a concentration of 0.5 mg/mL (Sigma-Aldrich Merck KGaA (Darmstadt, Germany). The plates were put back in the incubator, at 37 °C for 4 h, after which the obtained crystals were resuspended in acidic isopropanol. After brief mixing, the optic density was measured using a spectrophotometer at 570 nm.

### 4.4. Library Preparation and Bioinformatics Inspection

In order to obtain RNA from the cell pellets, we followed the manufacturer’s instructions of the Maxwell^®^ RSC simplyRNA Cells Kit (Promega, Madison, WI, USA). For the preparation of the library, TruSeq RNA Exome protocol (Illumina, San Diego, CA, USA) was used as already reported [39]. The library was analyzed using the Illumina instrument Miseq. The read quality was confirmed using fastqc (version 0.11.4, Babraham Institute, Cambridge, UK) and Trimmomatic (version 0.38, Usadel Lab, Aachen, Germany) [40] was used to remove poor quality reads and adapters. The cleaned reads were then aligned against the mouse reference genome GRCm39 and the annotation file version M28 using the Spliced Transcripts Alignment to a Reference (STAR, version 2.7.10a, New York, NY, USA) [41] and the final count was provided with htseq-count (version 0.6.1p1, European Molecular Biology Laboratory (EMBL, Heidelberg, Germany) [42]. Finally, the differentially expressed genes (DEGs) were obtained with the DESeq2 [43] Bioconductor [44] library of R (version 3.6.3, R Core Team).

### 4.5. Western Blot Analyses

The cells in the 6-well plate designated for Western Blot analyses underwent protein extraction using RIPA buffer. The protein concentrations were then assessed through Bradford Assay (Bio-Rad, Hercules, CA, USA), in order to load 25 µg of proteins per sample in gel wells. SDS-polyacrylamide gel electrophoresis (SDS-PAGE) followed the denaturation of the samples at 95 °C and then the proteins were transferred on PVDF membrane (Immobilon–P, Millipore, Burlington, MA, USA). The blocking was performed at room temperature for 1 h with 5% skimmed milk in TBS. For overnight incubation at 4 °C, the following antibodies were used:

Anti-CB1 (1:500, ThermoFisher Scientific, Rockford, IL, USA), anti-GAPDH HRP conjugate (1:1000, Cell Signaling, Danvers, MA, USA), anti-p-Akt (1:1000; Cell Signaling, Danvers, MA, USA), anti-Akt (1:1000, Cell Signaling, Danvers, MA, USA), anti-p-ERK1/2 (1:2000; Cell Signaling, Danvers, MA, USA), anti-ERK2 (1:1000; Cell Signaling, Danvers, MA, USA), anti-p-rps6 (1:1000; Cell Signaling, Danvers, MA, USA), anti-rps6 (1:1000; Cell Signaling, Danvers, MA, USA). After this incubation, membranes were incubated with the relative secondary antibodies for 1 h at room temperature: mouse anti-rabbit IgG-HRP (1:2000, Santa Cruz Biotechnology, Dallas, TX, USA, sc-2357) and chicken anti-mouse IgG (1:2000, ThermoFisher Scientific, Rockford, IL, USA). ChemiDoc™ MP System (Bio-Rad) was used for acquiring the bands after exposure to enhanced chemiluminescence system (Luminata Western HRP Substrates, Millipore, Burlington, MA, USA).

### 4.6. Statistical Analyses

GraphPad Prism 6.0 software (GraphPad Software, La Jolla, CA, USA) was used as tool for statistical analyses. According to the Kolmogorov–Smirnov test, the data fall into the normal distribution (α = 0.05) and the Brown–Forsythe test confirmed no different standard deviations through the samples (α = 0.05). Ordinary one-way ANOVA test was performed for purposes of multiple comparison. When the p-value was equal or less to 0.05, it was considered statistically significant. The results are expressed by mean ± SEM.

## 5. Conclusions

In order to understand the neurogenic properties of CBNR, this compound was administered to undifferentiated NSC-34 cells for 24 h at the doses 50, 20, 10, 7.5 and 5 µM. CBNR proved to not alter the vitality of cells. CBNR is able to interact with the CB1 receptor and 20 µM appears to be the optimal dose to induce neuronal differentiation. A 20 µM dose of CBNR can activate the Akt pathway through increasing its phosphorylation and, on the other hand, repressing the mTOR pathway and reducing ERK phosphorylation, as expected from CB1-mediated neuronal differentiation.

Our results indicate CBNR as a promising substance in the field of regenerative medicine in neuroscience. Further studies are needed to confirm these in vitro results with the results of in vivo models.

## Figures and Tables

**Table 1:** DEGs involved in neurogenesis/differentiation.

Gene	Ctrl vs. CBNR50	Ctrl vs. CBNR20	Ctrl vs. CBNR10	Ctrl vs. CBNR7.5	Ctrl vs. CBNR5
*Akt1*	0	0.14	0.23	0	0.09
*Bdnf*	0.91	1.16	0	0	0
*Capn1*	0.63	0.45	0	1.01	0.33
*Camk2b*	−1.18	−0.64	−1.06	−0.68	−0.89
*Cdc42*	−0.20	−0.25	0	0.30	0.10
*Cdk5r1*	0.36	0	0	0.63	−0.47
*Cdk5r2*	0.40	0.31	0.52	0.53	0
*Creb1*	0.18	0	0	0.42	0.19
*Limk1*	0.23	0	−0.28	−0.61	−0.20
*Map2*	0	0	0	0.91	0
*Mapk8*	0	0	0	0.35	0.28
*Nes*	−0.79	0.66	0	0	0
*Neurod1*	0	0	0	0.40	0
*Ppp3cb*	−0.15	0	0.16	0.25	0.14
*Ppp3cc*	0	0	0	0.40	0
*Rbfox2*	0	0	0.19	0.20	0.16
*Rock1*	−0.28	−0.22	−0.25	0	0
*Rock2*	−0.16	0	0	0.33	0.11
*Rps6*	−0.16	0	−1.27	−1.68	−1.44
*Syp*	0.52	0.66	0.46	−1.04	0.57
*Vim*	−0.39	−0.09	0	−0.11	0

The fold-change columns are based on log_2_(CBNRx/CTRL). The x in CBNRx is related to the different doses (50, 20, 10, 7.5, 5 µM). The values are rounded to the second decimal digit.

**Table 2:** DEGs involved in the cannabinoid receptor.

Gene	Ctrl vs. CBNR50	Ctrl vs. CBNR20	Ctrl vs. CBNR10	Ctrl vs. CBNR7.5	Ctrl vs. CBNR5
*Cnr1*	0.25	0.38	0.44	0.26	0.33
*Pparg*	0	−0.73	0	0.69	0
*Trpv2*	0	0	−1.14	0	−1.13

The fold-change columns are based on log_2_(CBNRx/CTRL). The x in CBNRx is related to the different doses (50, 20, 10, 7.5, 5 µM). The values are rounded to the second decimal digit.

## Data Availability

The data presented in this study are openly available in the NCBI Sequence Read Archive at BioProject, accession number PRJNA839187.
